# Maribavir for the Management of a Complex Cytomegalovirus (CMV) Infection After Liver Transplantation: Refractoriness and Intolerance to Standard Therapies in the Intensive Care Unit

**DOI:** 10.7759/cureus.114566

**Published:** 2026-08-15

**Authors:** Laura Santos, Hugo Inácio, Ana Cochicho Ramalho, Simão C Rodeia, Nuno Germano

**Affiliations:** 1 Intensive Care Unit, Unidade Local de Saúde de São José, Lisbon, PRT; 2 Intensive Care Unit, Unidade Local de Saúde Loures/Odivelas, Hospital Beatriz Ângelo, Lisbon, PRT

**Keywords:** antiviral refractoriness, antiviral toxicity, cytomegalovirus, intensive care unit, liver transplantation, maribavir

## Abstract

Managing cytomegalovirus (CMV) infection in the post-transplant setting is frequently complicated by severe adverse effects from standard antiviral therapy or viral resistance. We report the cases of two critically ill liver transplant recipients in whom maribavir, a kinase inhibitor approved for refractory/resistant CMV, was used.

In the first case, maribavir achieved rapid viral suppression in a 30-year-old patient with refractory CMV infection. In the second case, it served as effective salvage therapy, enabling bone marrow recovery following valganciclovir-induced toxicity while maintaining viral control.

Although both patients ultimately succumbed to non-CMV-related ICU complications, maribavir demonstrated clinical utility in achieving virological control, providing a crucial alternative for complex cases where conventional options are limited.

## Introduction

Cytomegalovirus (CMV) infection remains one of the most significant complications following solid organ transplantation, including liver transplantation, leading to substantial morbidity and mortality [1,2]. First-line antiviral therapies, such as (val)ganciclovir and foscarnet, although effective, are frequently limited by severe adverse effects or by the emergence of refractory/resistant CMV strains [3-5]. Refractory CMV infection is defined as persistence or an increase of >1 log_10_ in CMV DNA levels in blood or plasma despite at least two weeks of appropriately dosed antiviral therapy, whereas resistant CMV infection is defined by the presence of a genotypic mutation conferring reduced antiviral susceptibility; the two entities are not mutually exclusive and frequently coexist [6].

Critically ill liver transplant recipients pose distinct management challenges in this setting, as concurrent multi-organ dysfunction, including renal replacement therapy altering antiviral clearance, drug-induced bone marrow suppression limiting (val)ganciclovir use, and interactions with immunosuppressive regimens, frequently limits the use of standard first-line antiviral agents.

Maribavir is an antiviral drug approved for the treatment of CMV infections refractory or resistant to first-line therapies. It is a benzimidazole riboside that inhibits the UL97 protein kinase, specific to CMV. This mechanism prevents CMV DNA replication, encapsidation, and nuclear egress of viral capsids [7].

Although limited, the initial evidence suggests the drug is effective in reducing viral replication. The SOLSTICE trial [8] was a randomized study in patients undergoing solid organ or bone marrow transplantation with resistant/refractory CMV infection. It demonstrated the superior efficacy of maribavir when compared to investigator-assigned therapies, valganciclovir, ganciclovir, foscarnet, or cidofovir, in viral suppression and sustained symptomatic control. There was also a lower rate of treatment discontinuation related to adverse effects, namely nephrotoxicity and myelotoxicity. The AURORA trial [9] was a randomized controlled trial in which maribavir demonstrated a CMV viremia clearance rate at eight weeks not inferior to that of valganciclovir in the treatment of asymptomatic CMV infection after bone marrow transplantation. It also demonstrated better tolerability, with a lower rate of neutropenia and, consequently, a lower rate of treatment discontinuation.

The most common adverse effects are gastrointestinal symptoms, which are usually mild and, in most cases, do not limit the use of the drug [7].

Despite these promising trial results, real-world evidence regarding the use of maribavir in critically ill liver transplant recipients specifically remains scarce. To help address this literature gap, we report two clinical cases of liver transplant recipients in the ICU, illustrating how maribavir provided virological control in two distinct scenarios: refractoriness to first-line antiviral therapy (Case 1) and severe intolerance due to drug-induced toxicity (Case 2).

## Case presentation

Case 1: maribavir for refractory CMV infection

A 30-year-old female with a history of immune-mediated overlap syndrome was admitted due to acute liver failure of suspected toxin-induced or autoimmune aetiology. Following unsuccessful medical management (corticotherapy, N-acetylcysteine, and plasmapheresis), she underwent emergency liver transplantation, followed by re-transplantation three days later due to acute graft failure. Immunosuppression consisted of basiliximab, methylprednisolone, and tacrolimus. During admission to the ICU, she presented multiple complications, among which were acute kidney injury (Kidney Disease: Improving Global Outcomes (KDIGO) stage III, requiring renal replacement therapy (RRT)), suspected lupus flare with diffuse alveolar hemorrhage refractory to immunosuppression and plasmapheresis, *Clostridioides difficile* infection, and ventilator-associated pneumonia with no identified etiological agent.

**Figure 1:**
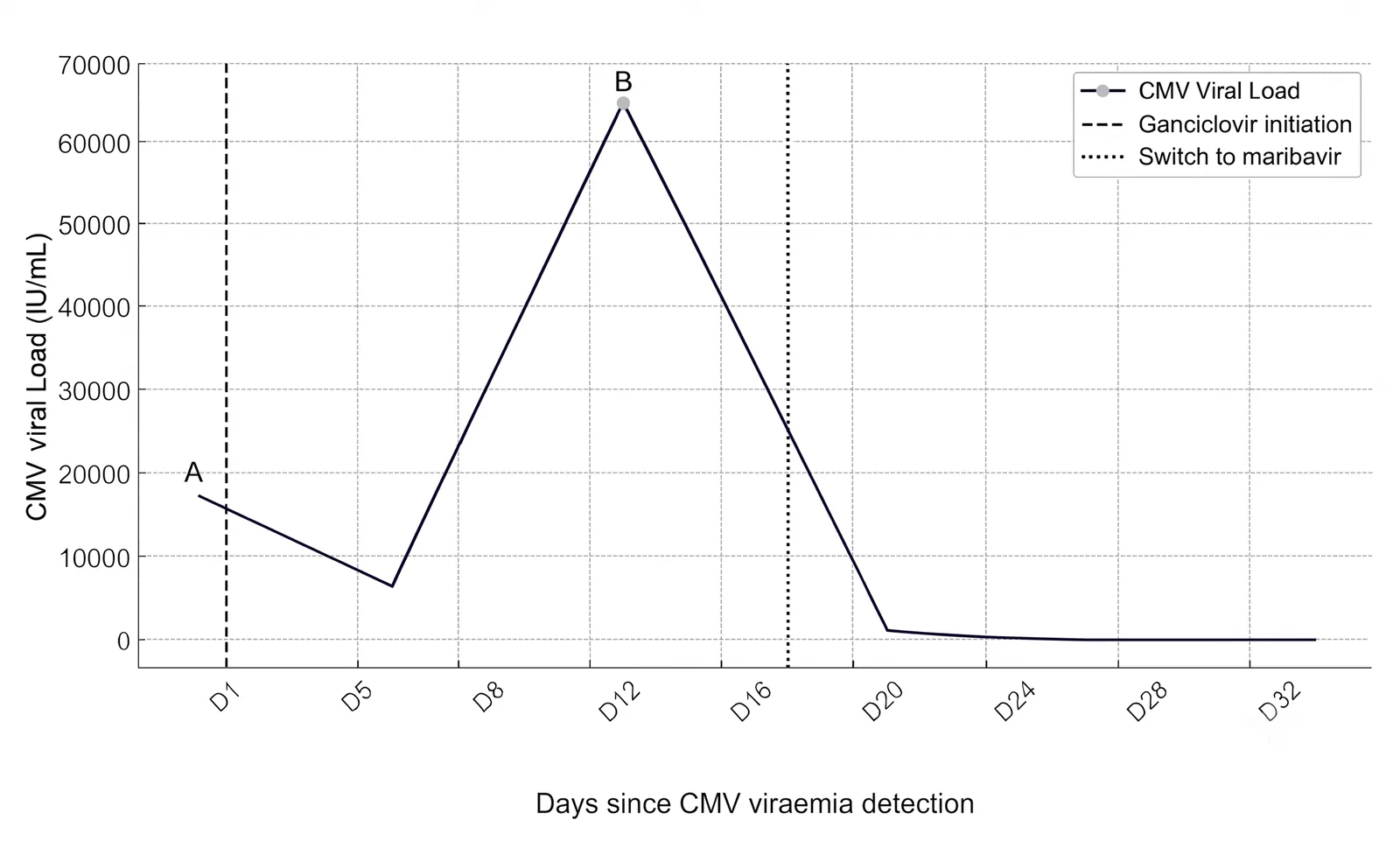
Progression of cytomegalovirus (CMV) viremia throughout admission in Case 1. A: Baseline viral load: 17,200 IU/mL (log_10_ 4.24); B: Peak viral load: 64,700 IU/mL (log_10_ 4.81); D: day

Her pre-transplant CMV serology indicated past infection (donor status unknown; recipient IgG positive, IgM negative). Two weeks after the second transplant, she presented with CMV reactivation with a baseline viremia of 17,200 IU/mL (log₁₀ 4.24; point A, Figure 1). Ganciclovir was initiated on day 2, adjusted for renal dysfunction, with RRT. Despite 11 days of appropriately dosed ganciclovir, viral load continued to rise, reaching a peak of 64,700 IU/mL (log_^₁₀^_ 4.81; point B, Figure 1) on day 13, at which point refractory CMV infection was clinically suspected. Given this lack of virological response, a decision was made to switch the antiviral to maribavir on day 17 (four days after refractoriness was suspected; 15 days after ganciclovir initiation). Maribavir was administered at the standard dose of 400 mg orally twice daily, with no dose adjustment based on renal function. A progressive reduction in CMV viral load was then observed, with viremia suppression achieved after 15 days of maribavir therapy (D32, Figure 1). Prior to the initiation of maribavir, a CMV resistance test had been requested (with results available only one month later), with no resistance-associated substitutions (RAS) detected for cidofovir, foscarnet, ganciclovir, maribavir, and letermovir (Table 1). Nausea and vomiting, attributable to maribavir, were observed, limiting progression of enteral feeding, but without severity to justify suspension of the drug.

**Table 1:** Cytomegalovirus (CMV) genotypic resistance profile for Case 1. RAS: resistance-associated substitutions

Generic name	Code	Associated genes	Detected RAS	Assessment
Cidofovir	CDF	UL54	(None)	Sensitive
Foscarnet	FOS	UL54	(None)	Sensitive
Ganciclovir	GCV	UL54, UL97	(None)	Sensitive
Letermovir	LMV	UL51, UL56, UL89	(None)	Sensitive
Maribavir	MBV	UL27, UL97	(None)	Sensitive

The patient had an unfavorable clinical course throughout the admission, culminating in death due to massive alveolar hemorrhage.

Case 2: maribavir as salvage therapy for severe antiviral toxicity

A 31-year-old female patient with a history of Cluster B personality disorder with psychotic symptoms medicated with paliperidone, lamotrigine, and olanzapine was admitted to the ICU with acute liver failure secondary to probable drug reaction with eosinophilia and systemic symptoms (DRESS) syndrome.

She underwent emergency liver transplantation on the third day of admission. Post-transplant immunosuppression included basiliximab, methylprednisolone, and tacrolimus, with the later addition of mycophenolate mofetil.

Taking into account the donor serology for CMV with positive IgM, she received one month of CMV prophylaxis, initially with ganciclovir, later transitioned to valganciclovir due to pancytopenia.

Her extended hospital admission was marked by multi-organ failure, including kidney injury requiring RRT and lower gastrointestinal bleeding from colonic ulcers consistent with DRESS-related pathology, alongside associated cardiomyopathy. Infectious challenges were equally severe, including Staphylococcus aureus bacteremia and septic shock secondary to soft tissue cellulitis, in addition to recurrent episodes of Candida glabrata ventilator-associated pneumonia with documented fungemia.

**Figure 2:**
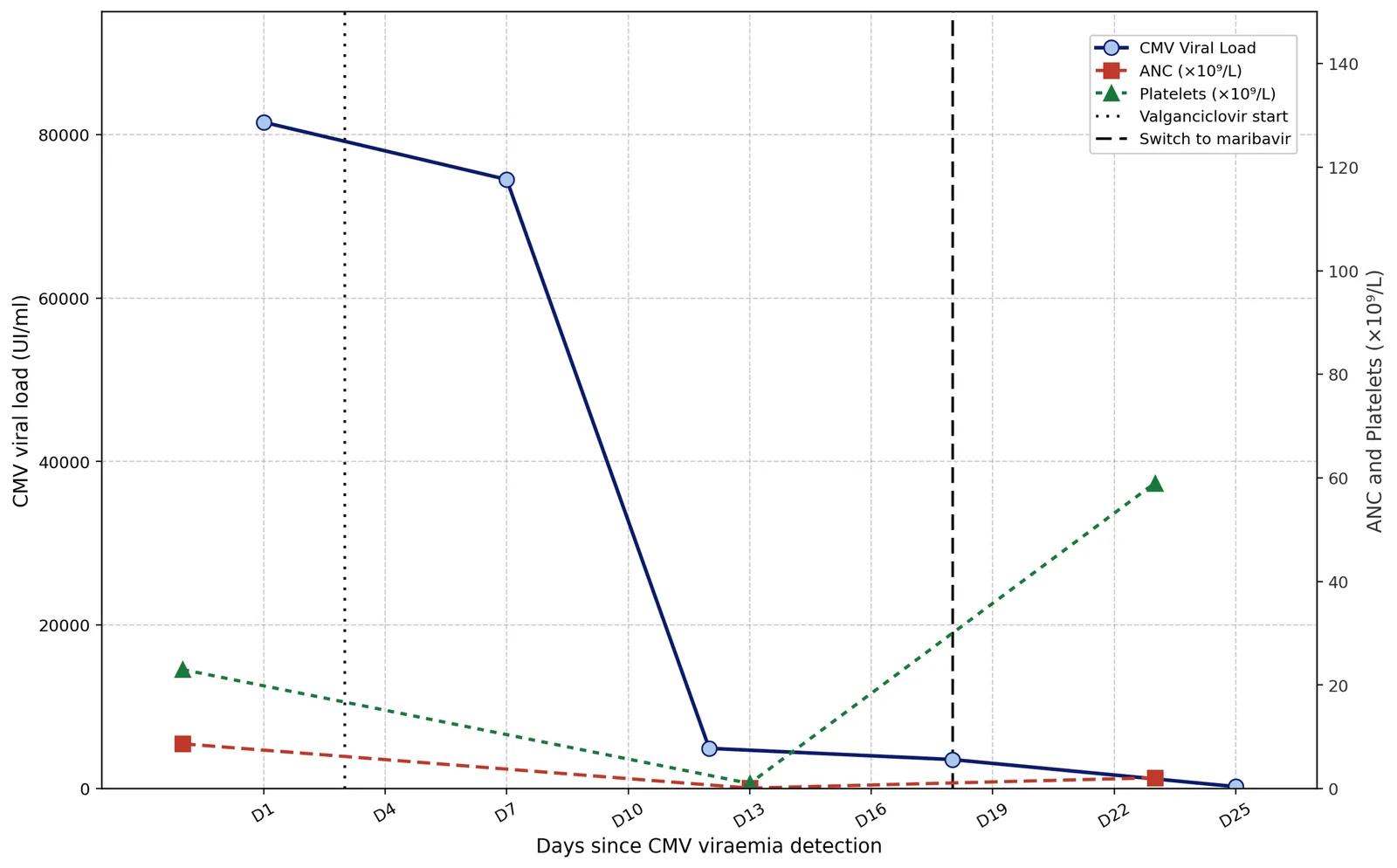
Progression of cytomegalovirus (CMV) viraemia and haematologic parameters (absolute neutrophil count (ANC), platelets) throughout admission in Case 2. D: day

During her third month of admission, she presented CMV viremia (81,500 UI/mL at detection, Figure 2), with proven ocular involvement confirmed by ophthalmologic examination demonstrating findings consistent with CMV retinitis. Central nervous system and bone marrow involvement were suspected but not formally confirmed: lumbar puncture and bone marrow aspirate could not be performed due to severe pancytopenia and bleeding risk, and cranial imaging (CT/MRI) was precluded by hemodynamic instability. She initiated valganciclovir on day 3, with a good virological response, the viral load declining to 4,930 UI/mL by day 12 and 3,560 UI/mL by day 18 (Figure 2), but it was complicated by severe pancytopenia: the baseline blood count prior to valganciclovir showed a white blood count (WBC) of 11.27×10⁹/L, an absolute neutrophil count (ANC) of 8.66×10⁹/L, and platelets of 23,000/µL, declining to a nadir on day 13 of WBC 0.3×10⁹/L, ANC 0.1×10⁹/L, and platelets 1,000/µL. Faced with profound bone marrow suppression making further (val)ganciclovir use unfeasible and a contraindication to foscarnet due to severe renal impairment [10], the multidisciplinary decision was to switch to maribavir as salvage therapy, despite its known limited penetration into the central nervous system and the eye [11]. Maribavir was initiated at the standard dose of 400 mg orally twice daily. During therapy with maribavir, CMV viral load continued its downward trajectory (244 UI/mL by day 25), and recovery of cell lineages was observed (Figure 2).

The patient had an unfavourable clinical course culminating in death due to refractory septic shock, prior to achieving full CMV viral clearance (D25, Figure 2).

## Discussion

The management of CMV infection in solid organ transplant recipients admitted to the ICU is highly challenging, particularly when complicated by multi-organ failure, drug toxicity, or viral refractoriness [1,2,5].

The two cases presented highlight the clinical utility of maribavir in these complex scenarios where traditional antiviral agents fail or are contraindicated.

In Case 1, the transition to maribavir resulted in a rapid and sustained suppression of the CMV viral load after refractoriness to first-line therapies was observed. This outcome aligns with the efficacy demonstrated in the SOLSTICE [8] and AURORA [9] trials. Notably, the absence of RAS underscores its viability as a second-line agent when first-line therapies fail, even without an identifiable genotypic cause [12].

Case 2 illustrates a different paradigm: maribavir as a salvage therapy in the context of severe drug-induced toxicity. The patient developed profound pancytopenia secondary to valganciclovir, a well-documented adverse effect that often leads to this therapy discontinuation [13]. With foscarnet contraindicated due to severe acute kidney injury requiring RRT, the clinical team was left without conventional options [10]. Although maribavir is not traditionally recommended for tissue-invasive CMV disease with ocular or suspected CNS involvement due to poor tissue penetration [11], its use was justified as a desperate measure to allow bone marrow recovery. Notably, the virological decline in Case 2 began during valganciclovir therapy, with viral load falling from 81,500 to 3,560 UI/mL before the switch to maribavir. The subsequent hematological recovery observed after the switch is therefore more plausibly attributable to valganciclovir withdrawal. Maribavir's role in this case should accordingly be framed as maintaining virological control while permitting bone marrow recovery, rather than as the direct cause of that recovery.

While both patients ultimately succumbed to non-CMV-related conditions and hemorrhagic ICU complications, strict antiviral objectives were successfully met. Maribavir demonstrated robust virological control in a refractory setting (Case 1) and provided a crucial safety bridge that allowed cellular recovery in a toxic setting (Case 2).

This report has several limitations. It describes only two cases from a single center, precluding any causal or comparative conclusions regarding maribavir's efficacy or safety. Therefore, findings should be interpreted as hypothesis-generating rather than confirmatory. The small sample size and heterogeneity of the two clinical scenarios further limit generalizability, and both patients' unfavorable outcomes were driven by non-CMV-related complications rather than treatment failure.

## Conclusions

These two cases suggest that maribavir may provide meaningful virological control in critically ill liver transplant recipients when standard antiviral therapies are ineffective (Case 1) or poorly tolerated due to myelotoxicity (Case 2). Its mechanism of action, directed at the viral UL97 kinase, was associated with sustained viral suppression in Case 1 and with continued virological control alongside hematological recovery in Case 2, without the nephrotoxicity or myelosuppression typically limiting first-line antiviral use.

However, these findings should be interpreted with caution. They derive from only two critically ill patients. Case 2 did not achieve full viral clearance prior to death; mild gastrointestinal adverse effects occurred in Case 1, and both patients ultimately died from non-CMV-related complications. Rather than establishing maribavir as a broadly effective and safe therapeutic alternative, these reports support its potential role as a salvage option in carefully selected, complex post-transplant patients, underscoring the need for larger, controlled studies to confirm its efficacy and safety in this critically ill population.
